# Carrier re-sequencing reveals rare but benign variants in recessive deafness genes

**DOI:** 10.1038/s41598-017-10099-2

**Published:** 2017-09-12

**Authors:** Longxia He, Xiuhong Pang, Penghui Chen, Xiaowen Wang, Tao Yang, Hao Wu

**Affiliations:** 1 Department of Otorhinolaryngology-Head and Neck Surgery, Shanghai Ninth People’s Hospital, Shanghai Jiaotong University School of Medicine, Shanghai, China; 2 Ear Institute, Shanghai Jiaotong University School of Medicine, Shanghai, China; 3 Shanghai Key Laboratory of Translational Medicine on Ear and Nose Diseases, Shanghai, China; 4 Department of Otolaryngology–Head and Neck Surgery, Xinhua Hospital, Shanghai Jiaotong University School of Medicine, Shanghai, China; 5 Department of Otorhinolaryngology-Head and Neck Surgery, Taizhou People’s Hospital, Jiangsu Province, China

## Abstract

For recessive Mendelian disorders, determining the pathogenicity of rare, non-synonymous variants in known causative genes can be challenging without expanded pedigrees and/or functional analysis. In this study, we proposed to establish a database of rare but benign variants in recessive deafness genes by systematic carrier re-sequencing. As a pilot study, 30 heterozygous carriers of pathogenic variants for deafness were identified from unaffected family members of 18 deaf probands. The entire coding regions of the corresponding genes were re-sequenced in those carriers by targeted next-generation sequencing or Sanger sequencing. A total of 32 non-synonymous variants were identified in the normal-hearing carriers *in trans* with the pathogenic variant and therefore were classified as benign. Among them were five rare (minor allele frequencies less than 0.005) variants that had previously undefined, disputable or even misclassified function: p.A434T (c.1300 G > A) in *SLC26A4*, p.R266Q (c.797 G > A) in *LOXHD1*, p.K96Q (c.286 A > C) in *MYO15A*, p.T123N (c.368 C > A) in *GJB2* and p.V1299I (c.797 G > A) in *CDH23*. Our results suggested that large scale carrier re-sequencing may be warranted to establish a database of rare but benign variants in causative genes in order to reduce false positive genetic diagnosis of recessive Mendelian disorders.

## Introduction

Hearing impairment, resulting from genetic, environmental and other causes, overall occurs in approximately 2–3‰ of children. Among them, an estimated 50–60% of cases can be attributed to genetic causes^1^. The genetic etiology of deafness is extremely heterogeneous. To date, over 80 genes have been reported to be associated with non-syndromic deafness (The hereditary hearing loss homepage, http://hereditaryhearingloss.org). With recent development and applications of targeted next-generation sequencing (NGS), the number of the reported variants for deafness has been drastically increased in just a few years^2–5^. On the other hand, it becomes imperative to determine the pathogenicity of the numerous non-synonymous variants identified from the deaf patients. In a recent study, for example, Shearer *et al*. re-categorized 4.2% of 2197 reported pathogenic variants for deafness as benign based on ethnic-specific differences in minor allele frequencies (MAFs)^6^. Conventional approaches to determine the pathogenicity of a variant include: (1) repeated documentations of the variant segregating with a disorder in various families; (2) detection of statistically significant differences in the allele frequency between large populations of cases and controls; and (3) extensive genotype–phenotype analyses^7–9^. Those approaches have been critical in providing a comprehensive understanding of correct variant prioritization, phenotypic outcome and clinical prognosis. This task, however, usually takes considerable time and effort to obtain the data. For rare variants with extremely low allele frequencies, it may be impossible to collect enough cases to complete such approaches. The task becomes even more challenging for recessive deafness, which accounts for approximately 80% of the genetic cases, as many such deaf patients do not have family history of deafness, limiting the use of intrafamilial genotype-phenotype co-segregation as an interpretation criterion^10^. Functional analysis, though convincing in some studies of specific deafness genes and variants, was not generally applicable in comprehensive diagnostic testing due to the large number of variants in question. Other criteria, such as evolutionary conservation analysis of the affected amino acids and computational prediction of the functional effect, provide suggestive rather than conclusive evidence.

Understanding of pathogenic variants is continuously changing in light of newly emerging large-scale population sequencing data. In this study, we proposed a new supporting strategy to identify the rare but benign variants by re-sequencing of the corresponding genes in carriers with known pathogenic variants. For fully penetrant disorders like most cases of recessive genetic deafness, any additional variant identified in the normal-hearing carriers *in trans* can be classified as benign.

## Results

### Recruiting of carriers with well-established pathogenic variants in recessive deafness genes

In previous studies, we have identified bi-allelic pathogenic mutations in recessive deafness genes in a number of deaf probands by targeted NGS^11^. Among these probands, 20 probands were selected who harbored bi-allelic, well-established pathogenic mutations. To qualify for the well-established pathogenic mutations, we chose the variants that were either previously reported with strong genetic evidence (such as linkage analysis, association study or functional study) or truncating variants (nonsense variants, frameshifting indels, etc) in genes in which similar truncating variants have been proven to be pathogenic. First- or second-generation normal-hearing relatives of the probands were then recruited and genotyped for the corresponding variants. From 18 such families, a total of 30 normal-hearing carriers (23 to 38 years old, mean age of 31.3 years) were identified with well-established pathogenic variants. Those pathogenic variants (bold in Supplementary Table S1) included 4 splice site variants, 10 nonsense variants, 8 frameshift variants and 3 missense variants in 11 recessive deafness genes.

### Carrier re-sequencing of the corresponding deafness genes

**Figure 1:**
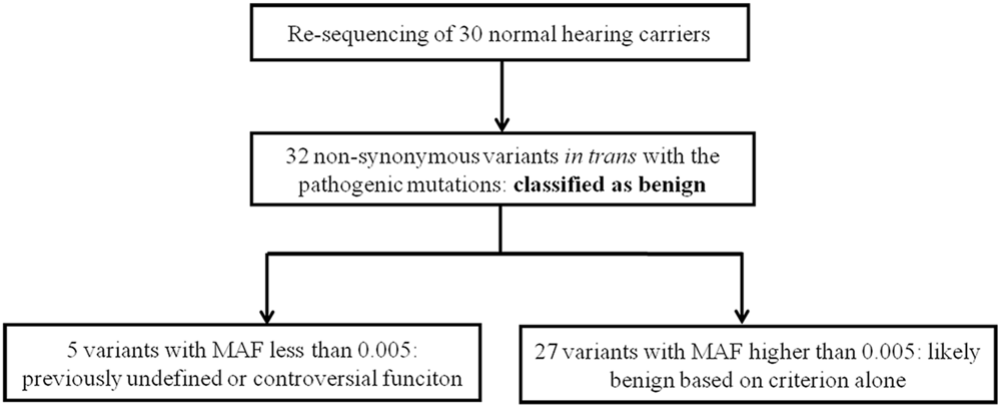
Workflow of the data analysis during carrier re-sequencing. These variants were presumed benign based on the family segregation data.

**Figure 2:**
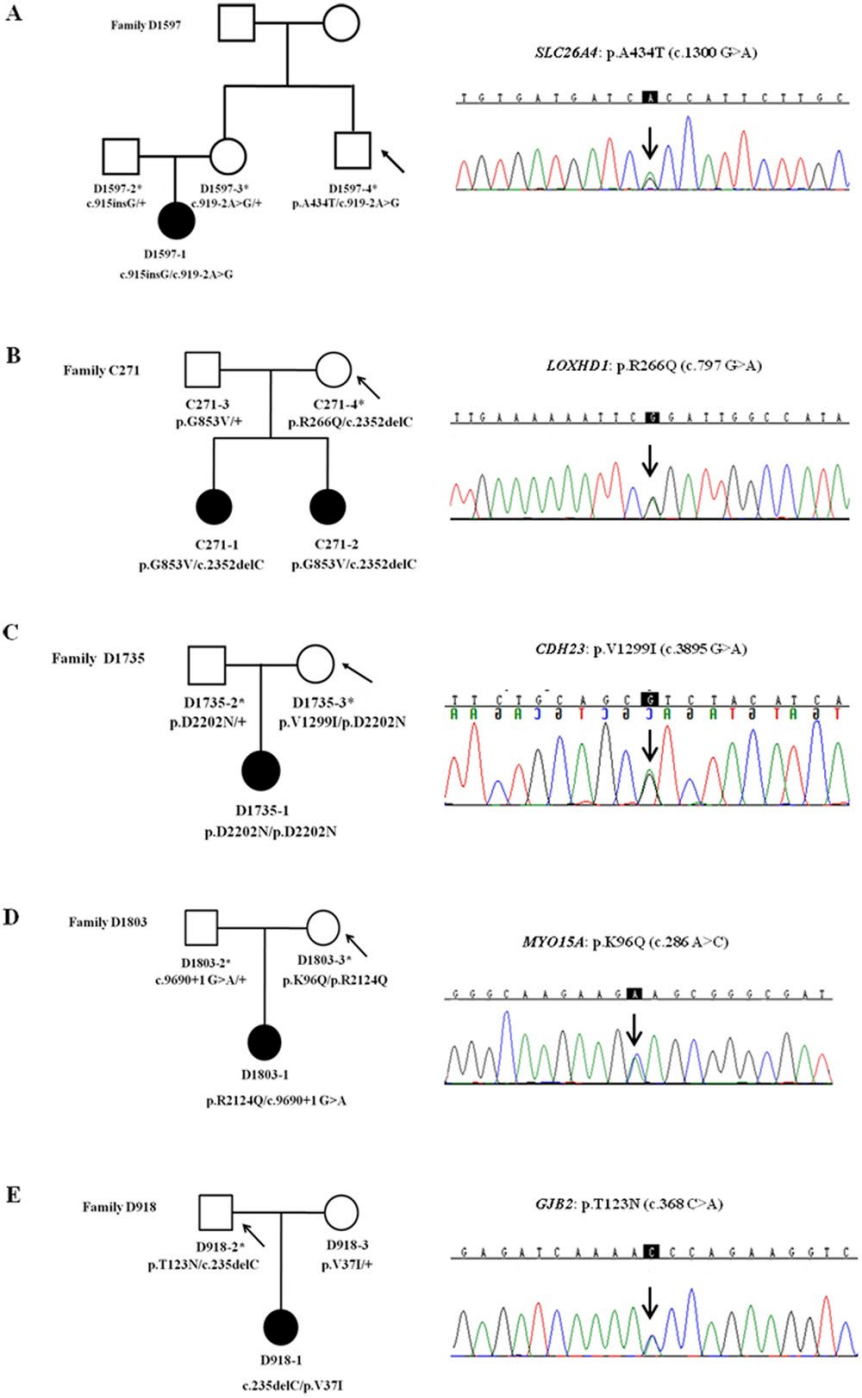
Family segregation and sequencing of the five rare, nonsynonymous variants. The normal-hearing carriers were marked by the asterisks. Carriers with the rare, non-synonymous variants were pointed by the arrows. The five rare, nonsynonymous variants are presumed benign based on the family segregation data.

Carrier re-sequencing of the corresponding deafness genes were then performed by Sanger sequencing or targeted NGS in all 30 carriers. The workflow of the data analysis is shown in Fig. 1. Based on family segregation analysis (Fig. 2 and data not shown), a total of 32 non-synonymous variants were identified *in trans* with the pathogenic variants (Supplementary Table S1). Since the carriers had normal hearing, those variants should be classified as benign.

**Figure 3:**
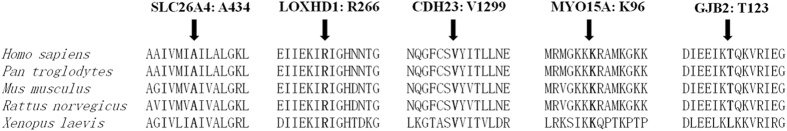
Multi-species sequence alignments of amino acids A434 in SLC26A4, R266 in LOXHD1, V1299 in CDH23, K96 in MYO15A and T123 in GJB2.

Among the 32 non-synonymous variants, 27 variants had MAF higher than 0.005, which rendered them likely benign by the MAF filtering criterion alone as suggested by Shearer *et al*.^6^. The remaining five variants, p.A343T in *SLC26A4*, p.R266Q (c.797 G > A) in *LOXHD1*, p.V1299I (c.797 G > A) in *CDH23*, p.K96Q (c.286 A > C) in *MYO15A* and p.T123N (c.368 C > A) in *GJB2*, had extremely low MAFs (<0.0007) in the NHLBI and GnomAD databases (Table 1) and changed a highly conserved amino acid (Fig. 3). As shown in Table 1, those five variants were either predicted to be pathogenic by computational tools such as Polyphen2, PROVEAN, SIFT or Mutation Taster, or documented as pathogenic in variant databases such as Deafness Variation Database (DVD), Human Genome Mutation Database (HGMD) or ClinVar. Those previously undefined, disputable or even misclassified function variants were examples whose function can be clarified as benign by the carrier re-sequencing approach.

**Table 1:** Rare, non-synonymous variants identified by carrier re-sequencing.

Variant (Reference transcript)	Gene	MAF (NHLBI)	MAF (GnomAD)	Polyphen2	PROVEAN (score)^a^	SIFT (score)^b^	Mutation Taster	DVD	HGMD	ClinVar
p.A434T (c.1300 G > A NM_000441)	*SLC26A4*	0	0.000024	benign	Neutral (−1.25)	Tolerated (0.209)	Disease causing	Pathogenic	Pathogenic	—
P.R266Q (c.797 G > A NM_001145472)	*LOXHD1*	0.000657	0.000241	Probably damaging	Deleterious (−3.27)	Damaging (0.02)	Disease causing	—	—	—
p.V1299I (c.797 G > A NM_022124)	*CDH23*	0.000397	0.000191	Possibly damaging	Neutral (−0.19)	Damaging (0.035)	Disease causing	Likely benign	—	Likely benign
p.K96Q (c.286 A > C NM_016239)	*MYO15A*	0	0.000013	Benign	Neutral (−1.59)	Damaging (0.001)	Disease causing	—	—	—
p.T123N (c.368 C > A NM_004004)	*GJB2*	0.000154	0.000464	Benign	Neutral (0.79)	Tolerated (0.545)	Polymorphism	Pathogenic	Deafness?	Benign /likely benign

^a^PROVEAN: Negative and positive scores indicate deleterious and neutral, respectively (cut-off score set at −2.5).

^b^SIFT: score ranges from 0 (deleterious) to 1 (neutral), divided by cut-off score (0.05).

### Clinical re-evaluation of the probands and carriers

For the five families (Fig. 2) with newly-clarified benign variants, we re-evaluated the clinical features of proband 1597-1, 271-1, D1735-1, D1803-1 and D918-1 who harbored known mutations in *SLC26A4*, *LOXHD1*, *CDH23*, *MYO15A* and *GJB2*, respectively. Consistent with previous genotype-phenotype correlation studies, all probands had prelingual, profound hearing loss. As expected for patients with pathogenic mutations in *SLC26A4*, inner ear CT scans showed that Proband D1597-1 with the c.919-2 A > G/c.915insG mutation in *SLC26A4* had enlarged vestibular aqueduct (EVA). As many Chinese Han non-syndromic EVA patients in early childhood, she did not have any thyroid abnormality resembling Pendred Syndrome at age 10 months. The corresponding carriers, D1597-4, D271-4, D1735-3, D1803-3 and D918-3 (Fig. 2), were all over 25 years old at the time of the test. Since most patients with recessive mutations in *SLC26A4*, *LOXHD1*, *CDH23*, *MYO15A* and *GJB2* had congenital or early-onset (<10 years) hearing loss, those carriers were deemed having true normal hearing phenotype. No signs of EVA, such as thyroid abnormality, were observed in carrier D1597-4.

## Discussion

In this study, we proposed a simple but effective approach to establish a database of rare but benign variants in recessive deafness genes by carrier re-sequencing. In previous studies, recessive pathogenic mutations have been identified in a large number of deaf patients worldwide. Many normal-hearing family members of those patients may carry a pathogenic variant in a given recessive deafness gene. Under the complete-penetrance mode for most reported recessive genetic deafness cases, any variants identified *in trans* with the pathogenic variants in the normal-hearing carriers can be classified as benign. In theory, sequencing of the corresponding genes in those carriers will generate a database of benign variants, which would be valuable for reducing the false positive diagnosis during genetic testing and counseling.

In this pilot study, we re-sequenced 30 carriers with well-established pathogenic variants in 11 recessive deafness genes. A total of 32 non-synonymous variants were identified *in trans* with the pathogenic variants (Supplementary Table S1) and were classified as benign by this carrier re-sequencing approach. Among them, five rare variants with previously undefined, disputable or even misclassified function were best examples to validate this approach (Table 1).

The p.A434T (c.1300 G > A) variant in *SLC26A4* was reported as a dominant or *de novo* pathogenic variant resulting in congenital sensorineural deafness and palmoplantar lichen in a previous study^12^. This variant was classified as pathogenic in the Deafness Variation Database (DVD) and the Human Gene Mutation Database (HGMD). The *LOXHD1* p.R266Q (c.797 G > A) and *MYO15A* p.K96Q (c.286 A > C) variants were novel. Both were predicted to be pathogenic by computational tools SIFT and Mutation Taster. The functional roles of the *GJB2* p.T123N (c.368 C > A) and *CDH23* p.V1299I (c.797 G > A) variants were disputable. The p.T123N (c.368 C > A) variant in *GJB2* was documented as “pathogenic” in DVD, “deafness?” in HGMD, but “benign/likely benign” in ClinVar. It was predicted as benign by all four computational tools but was reported as pathogenic by several previous studies^13, 14^. The p.V1299I (c.797 G > A) variant in *CDH23* was documented as “likely benign” in both DVD and ClinVar, but was classified as “unclassified variants (UV)” in the original report^15^, meaning it could not be confidently classified as either pathogenic or neutral based on a grading system recommended by the British Society of Human Genetics. It was predicted as possibly pathogenic by Polyphen2, pathogenic by SIFT and Mutation Taster. Combining the previous reports, variant database documentation, computational prediction and the extremely low MAFs (all less than 0.0007 in the GnomAD and NHLBI databases) of these five variants, our results showed that those functionally undefined, disputable or even misclassified function variants could be demonstrated as benign by the carrier re-sequencing approach. Our pilot study could serve as a blueprint for much larger-scale re-sequencing projects in the next stage and be expanded to other Mendelian genetic disorders.

## Materials and Methods

### Subjects

The deaf probands and the normal-hearing family members were recruited through Ear Institute, Shanghai Jiaotong University School of Medicine, Shanghai, China. All subjects signed a written informed consent to participate in this study. This study was approved by the Ethics Committee of Xinhua Hospital, Shanghai Jiaotong University School of Medicine and was in compliance with the Declaration of Helsinki.

### Auditory evaluation

Auditory evaluation was performed for all subjects including otoscope examination, tympanometry and pure-tone audiometry. The degree of hearing impairment was defined by the average level of hearing loss in the better ear for air pure-tone threshold in speech frequencies at 0.5, 1.0, 2.0, and 4.0 kHz. The severity of hearing impairment was calculated as mild (20–40 dB), moderate (41–70 dB), severe (71–95 dB) and profound (>95 dB). Normal hearing was defined as pure-tone thresholds less than 20 dB.

### Sequencing

The genomic DNA was extracted from whole blood. Sequencing of *GJB2* was completed by Sanger sequencing. Sequencing of all other deafness genes were completed by targeted NGS using the MyGenotics gene enrichment system (MyGenotics, Boston, MA, USA) and the Illumina HiSeq. 2000 sequencer (Illumina, San Diego, CA, USA) as previously described^16, 17^. Data analysis and bioinformatics processing were performed as previously described^18^. The Reads were aligned to HG19 using the BWA software and the variants were called using the Genome Analysis Toolkit (GATK), both with the default parameters. SNVs and indels were presented using Variant Call Format (VCF) version 4.1 and annotated using the ANNOVAR software. The 144 targeted deafness genes were listed in Supplementary Table S2. Among those 144 genes, only the 86 genes with recognized autosomal recessive inheritance were analyzed, while the remaining 58 genes with dominant and X-linked dominant/recessive inheritance were excluded from the current study.

### Computational analysis

MAFs of the variants were obtained from NHLBI (http://evs.gs.washington.edu/EVS/, 6503 samples) and GnomAD (http://gnomad.broadinstitute.org/, 123,136 samples) databases. Pathogenicity of the variants were predicted by computational tools PolyPhen2 (http://genetics.bwh.harvard.edu/pph2/), Mutation Taster (http://www.mutationtaster.org), PROVEAN and SIFT (http://sift.jcvi.org). Documentation of the pathogenicity of the variants was drawn from Deafness Variation Database (DVD v8.0, http://deafnessvariationdatabase.org/), Human Gene Mutation Database (HGMD, http://www.hgmd.cf.ac.uk/ac/index.php) and ClinVar (https://www.ncbi.nlm.nih.gov/clinvar/).

## Electronic supplementary material

Supplementary info
