# Supplementary material for: Carrier re-sequencing reveals rare but benign variants in recessive deafness genes
Source: Sci Rep. 2017 Sep 12;7:11355. doi: 10.1038/s41598-017-10099-2 (PMC5595904; doi:10.1038/s41598-017-10099-2)
Supplement: Supplementary file 1 — Supplementary info [file 41598_2017_10099_MOESM1_ESM.pdf]

Carrier re-sequencing reveals rare but benign variants in recessive deafness genes

Longxia He[1,2,3,4], Xiuhong Pang[5], Penghui Chen[1,2,3,4], Xiaowen Wang[1,2,3,4], Tao Yang[*1,2,3,4] and Hao Wu[*1,2,3]

[1]Department of Otorhinolaryngology-Head and Neck Surgery, Shanghai Ninth People's Hospital, Shanghai Jiaotong University School of Medicine, Shanghai, China

[2]Ear Institute, Shanghai Jiaotong University School of Medicine, Shanghai, China

[3]Shanghai Key Laboratory of Translational Medicine on Ear and Nose Diseases, Shanghai, China

[4] Department of Otolaryngology–Head and Neck Surgery, Xinhua Hospital, Shanghai Jiaotong University School of Medicine, Shanghai, China

[5]Department of Otorhinolaryngology-Head and Neck Surgery, Taizhou People's Hospital, Jiangsu Province, China

***Corresponding Authors:**

Tao Yang, Ear Institute, Shanghai Jiaotong University School of Medicine, Shanghai, China. Email: yangtfxl@sina.com, Tel: 86-21-25078893, Fax: 86-21-65152394

Hao Wu, Department of Otolaryngology-Head & Neck Surgery, Shanghai Ninth People's Hospital, Shanghai Jiaotong University School of Medicine, Shanghai, China. Email: wuhao622@sina.cn, Tel: 86-21-55570010, Fax: +86-21-65152394

Supplementary Table S1. Non-synonymous variants *in trans* with the pathogenic variants in the normal hearing carriers

Carriers	Gene	Reference transcript	Nucleotide change	Amino acid change	MAF (GnomAD)	MAF (NHLBI)
C212-2	*PTPRQ*	NM_001145026	**c.5426+1G>A**[*]	-	0	0
			c.2650T>C	p.F884L	0.3803	0.410206
C271-4	*LOXHD1*	NM_001145472	**c.2352delC**	**p.D784fs**	0	0
			c.1535A>G	p.E512G	0.08416	0.088042
			c.797G>A	p.R266Q	0.000241	0.000657
			c.130A>G	p.R44G	0.7675	0.771572
C310-1	*CDH23*	NM_022124	**c.3241C>T**	**p.R1081X**	0	0
			c.1469G>C	p.G490A	0.1998	0.121193
			c.4051A>G	p.N1351D	0.8159	0.812899
			c.4723G>A	p.A1575T	0.7752	0.741081
			c.6130G>A	p.E2044K	0.3111	0.265481
			c.7073G>A	p.R2358Q	0.3081	0.262008
			c.7139C>T	p.P2380L	0.2997	0.254335
			c.9373T>C	p.F3125L	0.06972	0.025518
D1335-1	*PCDH15*	NM_001142767	**c.4941_4942 insATCA**	**p.E1648fs**	0	0
			c.4853A>C	p.E1618A	0.2489	0.148641
			c.2675G>A	p.R892Q	0.2562	0.211133
			c.1027G>A	p.G343S	0.1656	0.234353
			c.55T>G	p.S19A	0.2181	0.216054
D1335-4	*PCDH15*	NM_001142767	**c.4941_4942 insATCA**	**p.E1648fs**	0	0
			c.4853A>C	p.E1618A	0.2489	0.148641
			c.2675G>A	p.R892Q	0.2562	0.211133
			c.1193A>C	p.D398A	0.238	0.131632
D1680-2	*MYO7A*	NM_000260	**c.3657dupC**	**p.G1219fs**	0	0
			c.47T>C	p.L16S	0.4348	0.468586
D715-2	*TECTA*	NM_005422	**c.990C>A**	**p.Y330X**	0	0
			c.1111A>G	p.R371G	0.403	0.497309
			c.2795T>C	p.V932A	0.2141	0.1984
			c.5171G>A	p.S1724N	0.9927	0.992079
D715-3	*TECTA*	NM_005422	**c.990C>A**	**p.Y330X**	0	0
			c.5171G>A	p.S1724N	0.9927	0.992079

C54-1	*PCDH15*	NM_001142767	**c.5506 C>T**	**p.Q1836X**	0.000008	0
			c.2675G>A	p.R892Q	0.2562	0.211133
			c.1193A>C	p.D398A	0.238	0.131632
D1735-2	*CDH23*	NM_022124	**c.6604G>A**	**p.D2202N**	0.000008	0
			c.4051A>G	p.N1351D	0.8159	0.812899
			c.4723G>A	p.A1575T	0.7752	0.741081
			c.5418C>G	p.D1806E	0.01445	0.012923
D1735-3	*CDH23*	NM_022124	**c.6604G>A**	**p.D2202N**	0.000008	0
			c.3895G>A	p.V1299I	0.000191	0.000397
			c.4051A>G	p.N1351D	0.8159	0.812899
			c.4723G>A	p.A1575T	0.7752	0.741081
F14-1	*TECTA*	NM_005422	**c.2719C>T**	**p.R907X**	0.000008	0
			c.5171G>A	p.S1724N	0.9927	0.992079
D1852-2	*OTOF*	NM_194322	**c. 4817 G>A**	**p.W1606X**	0	0
			c.62C>T	p.P21L	0.5512	0.411
D1852-3	*OTOF*		**c. 1273 C>T**	**p.R425X**	0.000016	-
			c.62C>T	p.P21L	0.5512	0.411
D1913-2	*TECTA*	NM_005422	**c.6250+2T>G**	-	0	0
			c.5171G>A	p.S1724N	0.9927	0.992079
D1913-3	*TECTA*	NM_005422	**c.6250+2T>G**	-	0	0
			c.1111A>G	p.R371G	0.403	0.497309
			c.2795T>C	p.V932A	0.2141	0.1984
			c.5171G>A	p.S1724N	0.9927	0.992079
D1597-4	*SLC26A4*	NM_000441	**c.919-2A>G**	**-**	0.000305	0
			c.1300G>A	p.A434T	0.000024	0
D1803-3	*MYO15A*	NM_016239	**c.6371G>A**	**p.R2124Q**	0	0.000078
			c.286A>C	p.K96Q	0.000013	0
			c.3413A>G	p.Q1138R	0.0142	0.014576
D1964-3	*MYO15A*	NM_016239	**c.3524dupA**	**p.S1176Vfs*14**	**0.0002**	0
			c.5929T>C	p.C1977R	0.7395	0.196594
			c.6052G>A	p.G2018R	0.2572	0.122405
D2055-3	*MYO15A*	NM_016239	**c.9315dupC**	**p.H3106Pfs*2**	0	0
			c.1783G>A	p.A595T	0.5289	0.335434
			c.2152T>G	p.W718G	0.6112	0

			c.5929T>C	p.C1977R	0.7395	0.196594
			c.6052G>A	p.G2018R	0.2572	0.122405
D2025-3	*MYO7A*	NM_000260	**c.3987C>A**	**p.Y1329X**	0	0
			c.4996A>T	p.S1666C	0.5440	0.410888
D2026-3	*MYO15A*	NM_016239	**c.8767C>T**	**p.R2923X**	0.000013	0.00008
			c.1783G>A	p.A595T	0.5289	0.335434
			c.2152T>G	p.W718G	0.6112	0
			c.5929T>C	p.C1977R	0.7395	0.196594
			c.6052G>A	p.G2018R	0.2572	0.122405
D918-2	*GJB2*	NM_004004	**c.235delC**	**p.L79Cfs**	0.0004	0
			c.368C>A	p.T123N	0.000464	0.000154
C212-3	*PTPRQ*	NM_001145026	**c.4090delA**	**p.K1364fs**	0	0
D1597-2	*SLC26A4*	NM_000441	**c.915insG**	**p.V306fs**	0.000016	0
D1597-3	*SLC26A4*	NM_000441	**c.919-2A>G**	**-**	0.000305	0
D1803-2	*MYO15A*	NM_016239	**c.9690+1G>A**	-	0	0
D1964-2	*MYO15A*	NM_016239	**c.4441T>C**	**p.S1481P**	0.000008	0
D1607-2	*ILDR1*	NM_001199799	**c.234C>A**	**p.Y78X**	0	0
D856-2	*MYO15A*	NM_016239	**c.8767C>T**	**p.R2923X**	0.00008	0

*The well-established pathogenic variants were marked in bold. The variants not in bold were *in trans* to the pathogenic variants and were presumed benign.

Supplementary Table S2. The 144 genes targeted by next-generation sequencing for deafness

ACTG1^{D}	*COL9A1*^{R}	*FGF8*^{D}	*HSD17B4*^{R}	*MYO15A*^{R}	*PROKR2*$^{D/R}$	*TBC1D24*$^{D/R}$
ALX3^{R}	*COL9A2*$^{D/R}$	*FGFR1*^{D}	*IL13*^{D}	*MYO1A*^{D}	*PRPS1*^{XLR}	*TCOF1*^{D}
BSND^{R}	*COMT2*^{R}	*FGFR3*^{D}	*ILDR1*^{R}	*MYO3A*^{R}	*PTPRQ*^{R}	*TECTA*$^{D/R}$
CABP2^{R}	*CRYM*^{D}	*FLNA*^{XLR}	*KARS*^{R}	*MYO6*$^{D/R}$	*RDX*^{R}	*TIMM8A*^{XLR}
CCDC50^{D}	*DFNA5*^{D}	*FOXI1*^{R}	*KCNE1*^{R}	*MYO7A*$^{D/R}$	*RPGR*^{XLD}	*TJP2*^{R}
CDH23^{R}	*DFNB31*^{R}	*FREM1*^{R}	*KCNJ10*^{R}	*NDP*^{XLR}	*SALL1*^{D}	*TMC1*$^{D/R}$
CEACAM16^{D}	*DFNB59*^{R}	*GATA3*^{D}	*KCNQ1*^{D}	*NF2*^{D}	*SALL4*^{D}	*TMIE*^{R}
CHD7^{D}	*DIABLO*^{D}	*GIPC3*^{R}	*KCNQ4*^{D}	*OTOA*^{R}	*SANS*^{R}	*TMPRSS3*^{R}
CIB2^{R}	*DIAPH1*^{D}	*GJB2*$^{D/R}$	*KRT9*^{D}	*OTOF*^{R}	*SEC23A*^{R}	*TNC*^{D}
CLDN14^{R}	*DIAPH3*^{D}	*GJB3*$^{D/R}$	*LAMA3*^{R}	*P2RX2*^{D}	*SEMA3E*^{D}	*TPRN*^{R}
CLPP^{R}	*DSPP*^{D}	*GJB6*$^{D/R}$	*LARS2*^{R}	*PABPN1*^{D}	*SERPINB6*^{R}	*TRIOBP*^{R}
CLRN1^{R}	*ECM1*^{R}	*GPR98*^{R}	*LHFPL5*^{D}	*PAX3*$^{D/R}$	*SIX1*^{D}	*TRMU*^{R}
COCH^{D}	*EDN3*$^{D/R}$	*GPSM2*^{R}	*LOXHD1*^{R}	*PCDH15*^{R}	*SIX5*^{D}	*TSPEAR*^{R}
COL11A1$^{D/R}$	*EDNRB*$^{D/R}$	*GRHL2*^{D}	*LRTOMT*^{R}	*PDZD7*^{R}	*SLC17A8*^{D}	*USH1C*^{R}
COL11A2$^{D/R}$	*ELMOD3*^{R}	*GRXCR1*^{R}	*MARVELD2*^{R}	*PNPT1*^{R}	*SLC26A4*^{R}	*USH1G*^{R}
COL2A1^{D}	*ESPN*^{R}	*HARS*^{R}	*MIR96*^{D}	*POLR1C*^{R}	*SLC26A5*^{R}	*USH2A*^{R}
COL4A3$^{D/R}$	*ESRRB*^{R}	*HARS2*^{R}	*MITF*^{D}	*POLR1D*^{D}	*SMPX*^{XLD}	*WFS1*^{D}
COL4A4^{R}	*EYA1*^{D}	*HGF*^{R}	*MSRB3*^{R}	*POU3F4*^{XLR}	*SNAI2*^{R}	*WHRN*^{R}
COL4A5^{XLR}	*EYA4*^{D}	*HMX1*^{R}	*MYH14*^{D}	*POU4F3*^{D}	*SOX10*^{D}	*NELL2*^{U}
COL4A6^{XLR}	*FGF3*^{R}	*HOXA2*$^{D/R}$	*MYH9*^{D}	*PROK2*^{D}	*STRC*^{R}	*KITLG*^{D}
COL1A1^{D}	*COL1A2*^{D}	*OTOG*^{R}	*OTOGL*^{R}			

D: autosomal dominant, n=48; R: autosomal recessive, n=69; D/R: autosomal dominant or recessive, n=17; XLD: X-linked dominant, n=2; XLR: X-linked recessive, n=7
